# Two Cases of Locomotor Ataxy Treated with Nitrate of Silver

**Published:** 1868-01

**Authors:** C. A. Hingston

**Affiliations:** London


### Two Cases of Locomotor Ataxy Treated with Nitrate of Silver.

BY C. A. HINGSTON, M. D., LONDON.

A sparely nourished, very intelligent, old-looking man, aged forty-four, residing in Plymouth, became a dispensary patient in January, 1866. He was quite blind and confined to his bed, and gave the following account of himself:—About four years ago, he first noticed a weakness of his sight, which gradually increased, until it disappeared first in the right eye and then in the left. His eyes were at the time examined with the aid of the ophthalmoscope, and he was told that the optic nerves were excavated, but without any other morbid condition. About eighteen months ago he began to suffer from shooting pains through his limbs and body, followed by slowly increasing weakness of the lower extremities, until recently he has been compelled wholly to keep to his bed.

The following were the symptoms observed:—His skin, pulse, tongue, bowels, and urine were apparently natural. He complained of constant pains of a shooting or darting character through the limbs and body; these pains were of equal severity during the day and night, and such as almost entirely to prevent sleep. There was almost complete loss of sensation of touch in the feet and legs, though temperature was well preserved. The muscular power of both limbs were very considerable, and he was able powerfully to resist flexion and extension, so much so as to render the production of those movements impossible without his consent.

On attempting to make him walk, the loss of co-ordination of movement was very marked; his legs flew about in all directions, and unless supported, he fell. He stated that the ground felt as if composed of round balls.

The case had been considered as one of paraplegia with rheumatism, and the ordinary remedies for that complaint had been administered without relief to the pain. Nitrate of silver was now given in quarter-grain doses three times daily, with the effect of producing immediate and complete cessation of pain. At the end of six weeks the remedy was left off, for fear of producing discoloration of the skin; and not only was he quite free from pain, but his power of walking had in some measure increased. Almost immediately, however, after leaving off the silver, the pain returned as severely as ever; and after a fortnight's interval, at his earnest request, the medicine was re-commenced, with again the effect of immediately curing the pain; and during the second six weeks he continued the treatment, he had not an hour's pain, though there was no further improvement in his power of walking. On once more stopping the silver, the pain again returned, though in a much mitigated form, so much so as to be quite bearable, and has not returned since with such severity as to require a renewal of treatment. When seen a few days since, he stated that the only time during his illness in which he had been quite free from pain was whilst taking the silver pills, and that though no essential improvement had taken place, yet the disease, which was previously steadily advancing, has since the commencement of treatment remained stationary.

The second case occurred in the wife of a laborer, aged thirty-nine, also residing in Plymouth. She came under treatment in June of last year, when the disease was in a much earlier stage than in the last instance. She stated that for the last two months she had been losing her power of vision, and was unable to see to read small print, or to do fine work. Soon afterwards she began to suffer from severe darting pains through her ankles and legs, not extending to the thighs. The pains were worse during the day than during the night, and were more severe in the right leg than the left; there was no tenderness to the touch; on the contrary, sensation was decidedly impaired in both legs, and she was unable to distinguish the number of fingers which touched her foot, even when they were placed very far apart from one another. She had been treated with alkalies and iodide of potassium, and all the acknowledged remedies for rheumatism, without effect.—The peculiar character and position of the pain, darting as it did through the calves of the legs, and not in the bones or joints, led to a suspicion of the commencement of ataxia, which was confirmed by the history of impaired vision. Nitrate of silver was at once administered, with the result of immediately curing the pain. After taking the pills for three weeks, she was well enough to leave off medicine; and though the pain slightly returned, it was not sufficient to inconvenience her, and her sight has not further deteriorated.

These cases are recorded as confirmatory of the conclusions which have been arrived at by other observers of the extreme value of nitrate of silver in the treatment of ataxia, not only in relieving the pains, by far the most harrassing of all the symptoms, but also in apparently arresting the nervous changes, and maintaining the disease *in statu quo.*—*Medical Times and Gazette.*

